# Gastric Microbiota in a Low–*Helicobacter pylori* Prevalence General Population and Their Associations With Gastric Lesions

**DOI:** 10.14309/ctg.0000000000000191

**Published:** 2020-07-27

**Authors:** Nelson Ndegwa, Alexander Ploner, Anders F. Andersson, Ulrika Zagai, Anna Andreasson, Michael Vieth, Nicholas J. Talley, Lars Agreus, Weimin Ye

**Affiliations:** 1 Department of Medical Epidemiology and Biostatistics, Karolinska Institutet, Stockholm, Sweden;; 2 Science for Life Laboratory, Department of Gene Technology, School of Engineering Sciences in Chemistry, Biotechnology and Health, KTH Royal Institute of Technology, Stockholm, Sweden;; 3 Division of Family Medicine, Karolinska Institutet, Stockholm, Sweden;; 4 Institute of Pathology, Bayreuth Clinic, Bayreuth, Germany;; 5 Faculty of Health and Medicine, HMRI University of Newcastle, New Lambton, Australia.

## Abstract

**INTRODUCTION:**

Non–*Helicobacter pylori* microbiota might account for some cases with unexplained chronic gastritis that may in a minority eventually progress to gastric cancer through the Correa cascade. We characterized gastric microbiota by describing the normal stomach, compared it with early precancerous lesions and other disease states, and assessed whether *H. pylori* status affects bacterial diversity.

**METHODS:**

In a population-based study of those with and without gastrointestinal symptoms, cytology brush samples were collected during endoscopy from 316 individuals. Mucosal status was classified as normal mucosa (171), nonatrophic *H. pylori* gastritis (33), atrophic gastritis (12), or antral chemical gastritis (61). The 16S rRNA gene sequencing and analysis were performed to characterize the microbiota.

**RESULTS:**

Microbiota in atrophic gastritis and nonatrophic *H. pylori* gastritis stomachs were dysbiotic and differed from those in the normal stomach (*P* = 0.001). The normal stomach had the highest microbial diversity, followed by antral chemical gastritis. The atrophic gastritis and chronic *H. pylori* gastritis groups had the lowest diversity, a difference that was statistically significant (*P* = 0.01). Besides *H. pylori*, non–*H. pylori* bacteria accounted for group differences. Microbial network analysis showed that the normal group network was most highly connected, whereas the *H. pylori* gastritis group had the lowest connection. We found an increasing positive co-occurrence of oral bacteria in the stomach because samples deviated from the normal network, some of which were pathogens. The *H. pylori–*negative group had the highest microbial diversity (Shannon index) compared with the *H. pylori*–positive group (*P* = 0.001).

**DISCUSSION:**

In this low–*H. pylori* prevalence general population, the gastric mucosal microbiota of the normal stomach differed significantly from those with nonatrophic or atrophic gastritis. There was an increasing abundance of pathogenic bacteria from the normal state to early precancerous states.

## INTRODUCTION

The human stomach, because of its harsh acidic environment, was largely considered a sterile environment until the discovery of *Helicobacter pylori* in 1982 (1), which provided a paradigm shift. Infection with *H. pylori* is the major cause of chronic gastritis, one of the most common lifelong serious diseases of the human stomach estimated to affect more than half of the world population (2). *H. pylori* gastritis may be nonatrophic or atrophic gastritis and in the latter form is characterized by the loss of normal mucosa glands in the antrum, corpus (and fundus), or both.

Corpus atrophy leads to impaired secretion of hydrochloric acid and intrinsic factor that places the patients at risk for malabsorption of vitamin B_12_ and some macronutrients. The hypochlorhydric stomach also becomes more favorable to colonization by oral microbiota, some of which are capable of producing carcinogens, such as acetaldehyde and nitrosamines (3,4). Although the risk of gastric cancer in people with normal/healthy stomach mucosa without *H. pylori* colonization is extremely low, it doubles in patients with nonatrophic *H. pylori* chronic gastritis (5) and rises exponentially with the progression to atrophic gastritis (6).

Although half of the world population is colonized by *H. pylori*, relatively few develop gastric cancer. Furthermore, populations vastly differ by *H. pylori* prevalence (2), with most Western populations having a low prevalence. In a Swedish study (7), a low–*H. pylori* prevalent population, an increase in atrophic gastritis among young adults was observed, and from the same population, it was reported that these individuals have an excess risk of developing gastric cancer compared with the general population (8).

Such observations lend support to the hypothesis that non–*H. pylori* microbiota might also be involved in the development of chronic gastritis with the eventual progression to gastric cancer through the multistep Correa cascade (3). Studies identifying dysbiosis (9) in the precancerous stomach (10) and limited gastric cancer animal models are also consistent with this hypothesis (11). With the increase in the incidence of gastric cancer in younger people in some parts of the world where *H. pylori* is declining (12,13), an understanding of the role of the non–*H. pylori* gastric microbiota in disease needs to be a priority.

Previous gastric microbiota studies have been hospital based in study design and/or had a small sample size, limiting generalizability (14–17). Moreover, different studies have made use of different sources of samples, such as gastric juice or tissue biopsy (14,15,18) or feces (19), have been conducted in populations with markedly different *H. pylori* prevalence rates (10,14–16), and have used reference groups, such as chronic gastritis not the healthy stomach (14,15). These factors affect comparability between studies, and although the early findings have been pivotal to our appreciation of the role of bacteria in the stomach, it is necessary to further expand and confirm the observations in larger studies with a population-based study design. In this study, we collected cytology brush samples during upper endoscopy with the aim of characterizing gastric non–*H. pylori* microbiota in a population-based cohort of 316 individuals from a low–*H. pylori* prevalence general population to (a) describe the mucosa-associated microbiome in the normal stomach, (b) compare the healthy stomach microbiota with early precancerous lesions and other disease states, and (c) assess whether *H. pylori* status affects bacterial diversity.

## METHODS

### Study population and sample collection

This study is part of the LongGerd project, a longitudinal population-based study of gastrointestinal symptoms conducted in Sweden, with surveys in 1988, 1989, and 1995, that have been described previously (20,21). A follow-up study was set up in 2011–2012 described in detail elsewhere (22) and forms the basis for this project. Briefly, a series of surveys of abdominal/gastrointestinal symptoms were conducted in the municipality of Östhammar, Sweden. In January 2012, a follow-up project was set up and completed in April 2012 that involved residents of the municipality ages 20 years or older who were born on the 3rd, 12th, or 24th of each month. In total, 1,842 eligible subjects were asked to fill in several mailed questionnaires, and 1,034 participants who responded to the mail and were 79 years or younger were invited to endoscopy (with biopsies) and blood sampling. Eventually, 388 subjects aged between 20 and 79 years (203 women and 185 men, with a mean age of 54 years) underwent endoscopy examination and blood sample collection. The exclusion criteria included contraindications, older than 80 years, and those who could not read the mailed Abdominal Symptoms Questionnaire because it was written in Swedish. Detailed participant inclusion/exclusion criteria are illustrated in Figure 2 of an earlier published article (22) (see Figure S0, Supplementary Digital Content 1, http://links.lww.com/CTG/A307). A structured endoscopic protocol was used, and the findings registered according to the current standards (23). All procedures were video recorded for second opinion and consensus sessions. Gastric biopsies were taken from the cardia 2 cm below the gastroesophageal junction, the corpus, antrum, and any visible aberration, with 2 biopsies from each location. Biopsies were locally stored in a −80°C freezer and transported deep-frozen to the Department of Pathology, Klinikum Bayreuth, Germany, where histopathology was analyzed. Blood samples were also collected and tested for antibodies against *H. pylori* and gastric atrophy markers (gastrin-17 and pepsinogen I/II) and detected by an enzyme-linked immunosorbent assay kit (GastroPanel, Biohit Plc, Helsinki, Finland). In addition, 2 endoscopic cytology brushing samples from the corpus and antrum were collected and stored in −80°C freezer.

### DNA extraction and sequencing

DNA was extracted from endoscopy brushes by following the Mag Maxi Manual protocol of DNA Isolation Kit, Cat. No. 40403, LGC Genomics GmbH (Germany). Primers 341F (CCTACGGGNGGCWGCAG) and 805R (GACTACHVGGGTATCTAATCC) (24) targeting the V3-V4 regions of the bacterial 16S rRNA gene generated PCR amplicons using KAPA HiFi HotStart ReadyMix (2X) (KAPA Biosystems, Kit Code KK2602) in replicates. Replicates were pooled and barcoded using dual indexing primers in a second PCR amplification. Libraries were sent to the National Genomics Infrastructure/Science for Life Laboratories, Stockholm, for sequencing on Illumina MiSeq (Illumina Inc) using a 2 × 300-bp paired-end protocol (MiSeq Reagent Kits v3) (see Supplementary Results, Supplementary Digital Content 1, http://links.lww.com/CTG/A307).

### Reads processing

Reads were processed following Uparse pipeline (25) with slight modifications. Usearch was used to trim off low-quality bases on the 3′ end, forward and reverse primers before merging reads to a minimum length of 350 bp. PhiX sequences were removed. Poor quality reads (with >1 expected errors) were removed. Operational taxonomic units (OTUs) were clustered at 97% sequence identity excluding singletons. Chimeric sequences were removed using Uchime. OTU taxonomy assignment was performed using RDP database (26). Finally, OTU and taxonomy tables were generated and used for further analysis in R.

### Sample grouping

Participants were *a priori* grouped into 5 main groups based on serology and histology: normal/*H. pylori*–negative gastritis, *H. pylori* gastritis without corpus atrophy, corpus atrophic gastritis, antral chemical gastritis, and post–*H. pylori* eradication/seropositive group. Those with other types of gastritis or those that could not be classified into the above-mentioned groups were excluded (see Supplementary Results, Supplementary Digital Content 1, http://links.lww.com/CTG/A307).

### Statistical analysis

Sample data, OTU table, and phylogenetic tree were integrated into one object using Phyloseq (27). The data set was rarefied to 3,000 as the minimum count after prevalence filtering. Ordination and permutational multivariate analysis of variance (PERMANOVA) analyses were performed using Vegan package (28). Differential abundance testing was performed using DESeq2 (29) with unrarefied data. All the previously mentioned analyses were conducted in R (30). Microbial co-occurrence network analysis was performed using SparCC (31) and visualized in Gephi (32). Microbial function prediction was performed using Picrust (33) and analyzed in STAMP software (34) and the results presented in the Supplementary Results (see Supplementary Digital Content 1, http://links.lww.com/CTG/A307).

### Co-occurrence network analysis

We used SparCC to calculate correlations between OTU abundances in the microbiota data while accounting for their inherent sparsity and compositionality (31) (see Supplementary Results, Supplementary Digital Content 1, http://links.lww.com/CTG/A307). Identification of oral bacteria was performed by comparing OTU representative sequences against the Human Oral Microbiome (HOMD 16S rRNA RefSeq Version 15.1) database (35), and sequences with 100% identity match were considered of oral origin.

### Ethics

Approval for the study was obtained from the Ethics Committee of the Uppsala University (Dnr 2010/443).

## RESULTS

The baseline characteristics of study participants are presented in Table 1. We sequenced 316 of 388 (81%) corpus cytology brushing samples and 318 of 388 (82%) antrum samples. The remaining samples either had little DNA or did not yield enough material for sequencing. Amplicon sequencing yielded an average of 29,030 (range: 11–198,812) reads per sample. Quality filtering was performed to obtain high-quality nonchimeric reads that were clustered at 97% identity into 737 nonsingleton OTUs. We included both positive (see Figures S1 and S2, Supplementary Digital Content 1, http://links.lww.com/CTG/A307) and negative control (see Figures S3 and S4, Supplementary Digital Content 1, http://links.lww.com/CTG/A307) samples. Furthermore, OTUs with ambiguous phylum annotations (missing or uncharacterized) or those with prevalence less than 3.8% of the total samples (6 samples) were filtered out leaving 9,157,556 (99.8%) reads, with each sample having an average of 28,980 (range: 11–198,790) reads and 434 OTUs in the filtered data set that was used for further analysis. Microbiota in the stomach anatomical sites of the corpus and antrum were found to be similar in alpha (see Figure S5, Supplementary Digital Content 1, http://links.lww.com/CTG/A307; observed [*P* = 0.24], Shannon [*P* = 0.17]) and beta diversity estimates (PERMANOVA [*P* = 0.05], Bray–Curtis distances, principal coordinates analysis [PCoA] plot; see Figure S6, Supplementary Digital Content 1, http://links.lww.com/CTG/A307).

**Table 1.**
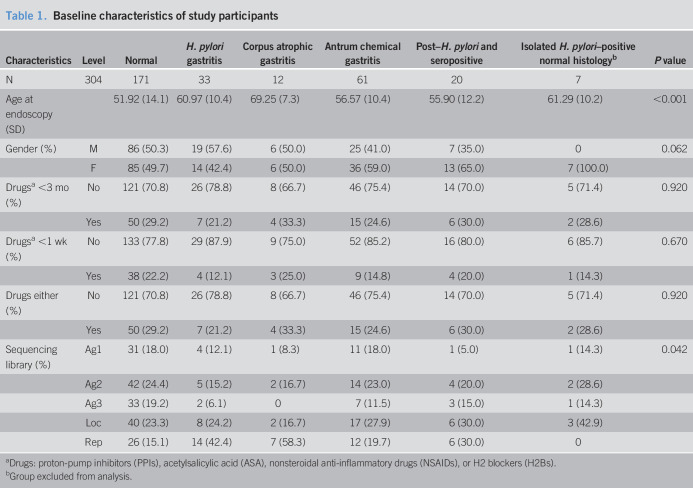
Baseline characteristics of study participants

### Normal stomach microbiota

**Figure 1.**
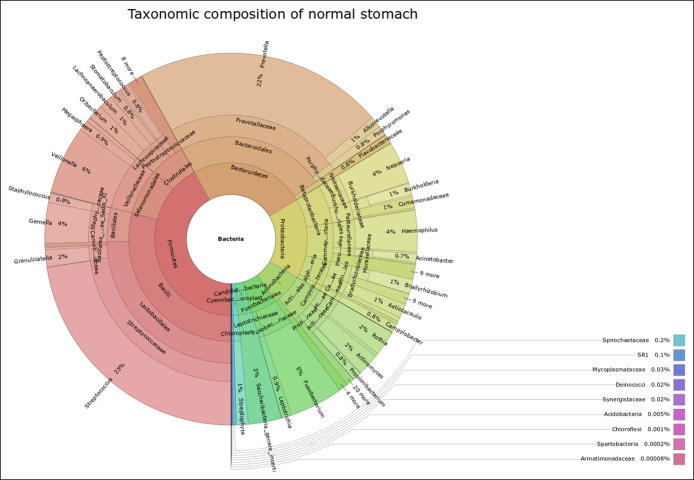
A Krona plot showing the bacterial taxonomic composition at different levels of the normal stomach. The concentric circles start from the kingdom level (innermost circle) to the genus level (outermost circle).

OTUs in the normal stomach samples were classified into 16 bacterial phyla, with 9 phyla each constituting less than 1% of the total abundance. The 5 most abundant phyla accounting for 96% of the total reads in the normal stomach samples were Firmicutes (42%), Bacteroidetes (24%), Proteobacteria (17%), Actinobacteria (7%), and Fusobacteria (6%). Seventy-six families were identified within the normal stomach group with the 5 most abundant families (60% of families) being Streptococcaceae (23%), Prevotellaceae (23%), Veillonellaceae (7%), Bacillales incertae sedis XI (4%), and Lachnospiraceae (3%). At the genus level, 140 genera were identified in total within the normal stomach group of which *Streptococcus* (23%), *Prevotella* (22%), *Veillonella* (6%), *Fusobacterium* (5%), *Gemella* (4%), *Neisseria* (4%), and *Haemophilus* (4%) constituted the 7 top genera (68% of all genera) (Figure 1).

### The stomach in normal state is more diverse than that in other states

**Figure 2.**
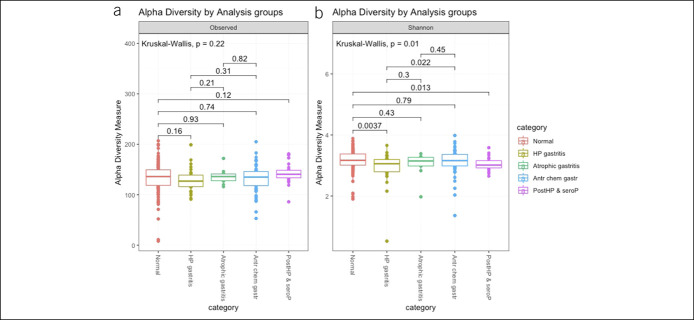
Alpha diversity box plots of the analysis groups using observed and Shannon index diversity measures.

Analysis of the community structure using the Shannon index suggested that the normal group had the highest microbial diversity (3.16), followed by antral chemical gastritis (3.12), whereas the atrophic gastritis (3.04) and *H. pylori* gastritis (2.94) groups had the lowest alpha diversity. The overall group difference was statistically significant (*P* = 0.01) (Figure 2).

### Diversity between groups

**Figure 3.**
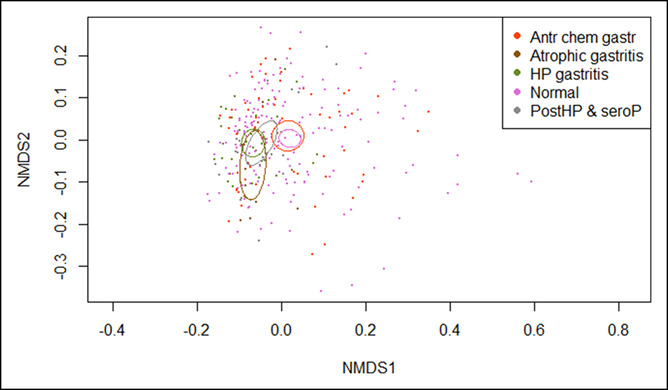
Plot of Bray–Curtis distances based on nonmetric multidimensional scaling (NMDS) (stress = 0.086). The ellipses represent 95% confidence intervals surrounding each group.

A clear separation of normal and antral chemical gastritis samples from *H. pylori* gastritis and atrophic gastritis samples was seen using the indirect ordination method of nonmetric multidimensional scaling with Bray–Curtis dissimilarity metric (Figure 3). Similar results were obtained with direct ordination using canonical correspondence analysis (see Figure S10 to S13, Supplementary Digital Content 1, http://links.lww.com/CTG/A307). The results from the above ordination-based analyses were similar after the removal of the OTU belonging to the genus *Helicobacter*.

PERMANOVA was used to test whether samples differed between the groups (normal, atrophic gastritis, *H. pylori* gastritis, and antrum chemical gastritis) while adjusting for age at endoscopy and batch effects (sequencing libraries). The distribution and abundance of microbiota composition were significantly different (*P* = 0.001, permutations = 999, R^2^ = 6%) between the groups.

### Differential abundance testing

We conducted the differential analysis using DESeq2 on raw prevalence filtered data set to identify bacteria responsible for separation of the different groups at the phylum and genus levels. DESeq2 uses a negative binomial distribution model to test for differences in read counts between the 2 defined groups and further controls for false discovery rate using the Benjamini and Hochberg procedure.

**Figure 4.**
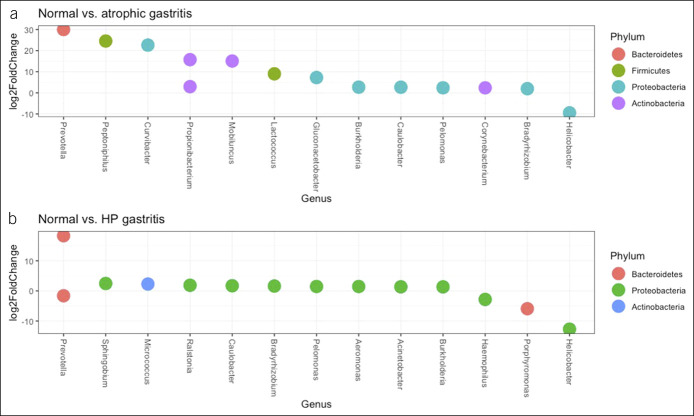
Differentially enriched operational taxonomic units between normal–atrophic gastritis (**a**) and normal–*H. pylori* gastritis (**b**) at the genus and phylum levels. These operational taxonomic units were among the most significantly differentially abundant (alpha = 0.05, after multiple testing correction using the Benjamini–Hochberg method) between the groups shown in the title of the respective plot.

Several genera demonstrated significant abundance differences between the groups while adjusting for age at endoscopy and batch effects (sequencing libraries) (Figure 4 and see Table S1, Supplementary Digital Content 2, http://links.lww.com/CTG/A308). We identified genera that were differentially abundant in at least 2 of the grouping categories and found 14 genera that had dissimilar abundances between normal–atrophic gastritis group comparison, 14 genera between normal–*H. pylori* gastritis groups, 20 genera between atrophic gastritis–antral chemical gastritis groups, and 16 genera between *H. pylori* gastritis–antral chemical gastritis group comparison.

Unsurprisingly, the *Helicobacter* genus was a major component of the differences between all the groups. However, other genera contributed to the differences: normal–atrophic gastritis (*Bradyrhizobium*, *Burkholderia*, *Caulobacter*, *Corynebacterium*, *Curvibacter*, *Gluconacetobacter*, *Lactococcus*, *Mobiluncus*, *Pelomonas*, *Peptoniphilus*, *Prevotella*, and *Propionibacterium*) and antrum chemical gastritis–atrophic gastritis (*Acinetobacter*, *Aeromonas*, *Asticcacaulis*, *Atopobium*, *Delftia*, *Ewingella*, and *Kocuria* in addition to those identified in normal–atrophic gastritis comparison). The *H. pylori* gastritis–normal/antrum chemical gastritis group comparisons showed similar differentially enriched genera (*Acinetobacter*, *Aeromonas*, *Bradyrhizobium*, *Burkholderia*, *Caulobacter*, *Helicobacter*, *Micrococcus*, *Pelomonas*, *Ralstonia*, and *Sphingobium*) except for the following: *H. pylori* gastritis–antrum chemical gastritis (the genera *Brevibacterium*, *Delftia*, *Halomonas*, *Kocuria*, *Lactococcus*, and *Variovorax* were differentially enriched only within this comparison), whereas for the *H. pylori* gastritis–normal group comparison, *Haemophilus*, *Porphyromonas*, and *Prevotella* were differentially enriched in this comparison. The following groups were similar because there were no statistically significant differences between them: normal–antrum chemical gastritis, atrophic gastritis–*H. pylori* gastritis, and antrum chemical gastritis–post–*H. pylori* eradication and seropositive groups.

### Microbial co-occurrence network analysis

To explore the interactions between microbes and environmental effects on their coexistence within biological communities (36), we performed a network analysis to detect co-occurrence patterns between taxa within the normal, *H. pylori* gastritis, and atrophic gastritis groups. The normal group network shown in Figure S14 (see Supplementary Digital Content 1, http://links.lww.com/CTG/A307) (with 330 nodes and 268 edges, 41 modules after filtering) had 4 communities with the highest connection (average degree = 11.17, average path length = 1.94) compared with the *H. pylori* gastritis and atrophic gastritis groups.

The *H. pylori* gastritis group network, shown in Figure S15 (see Supplementary Digital Content 1, http://links.lww.com/CTG/A307) (with 127 nodes and 44 edges, 24 modules after filtering), had 4 communities but with the lowest connection (average degree = 3.67, average path length = 2.83) compared with the other groups. The atrophic gastritis group network, shown in Figure S16 (see Supplementary Digital Content 1, http://links.lww.com/CTG/A307) (with 92 nodes and 148 edges, 56 modules after filtering), had 2 communities with medium connection (average degree = 5.29, average path length = 2.48) compared with the other groups. The strongest positive and negative associations between the OTUs at the genus level within the 3 groups were also identified (see Supplementary Results, Supplementary Digital Content 1, http://links.lww.com/CTG/A307, Table S2, Supplementary Digital Content 3, http://links.lww.com/CTG/A309, Tables S3a, S3b, and S4, Supplementary Digital Content 4–6, http://links.lww.com/CTG/A330, http://links.lww.com/CTG/A331, http://links.lww.com/CTG/A332).

### *H. pylori* status and microbiota diversity

**Figure 5.**
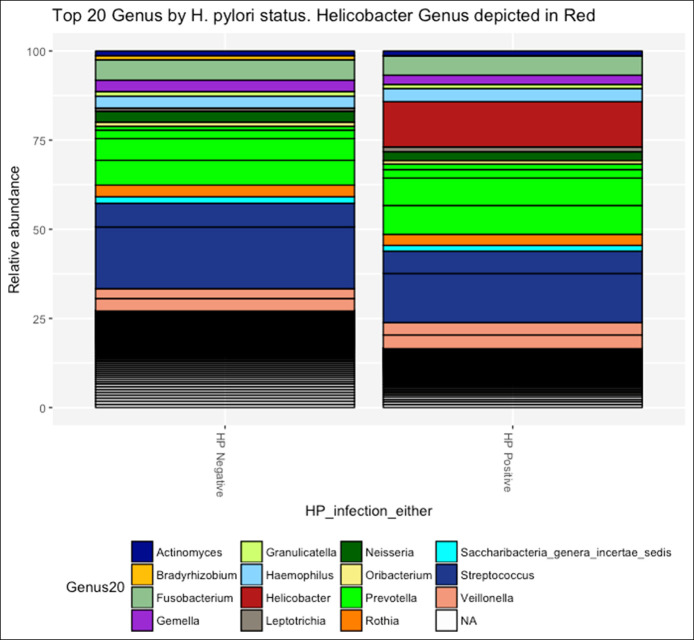
Proportion of *H. pylori* reads by *H. pylori* status. *H. pylori* negative = 0.04% and *H. pylori* positive = 12.71%.

**Figure 6.**
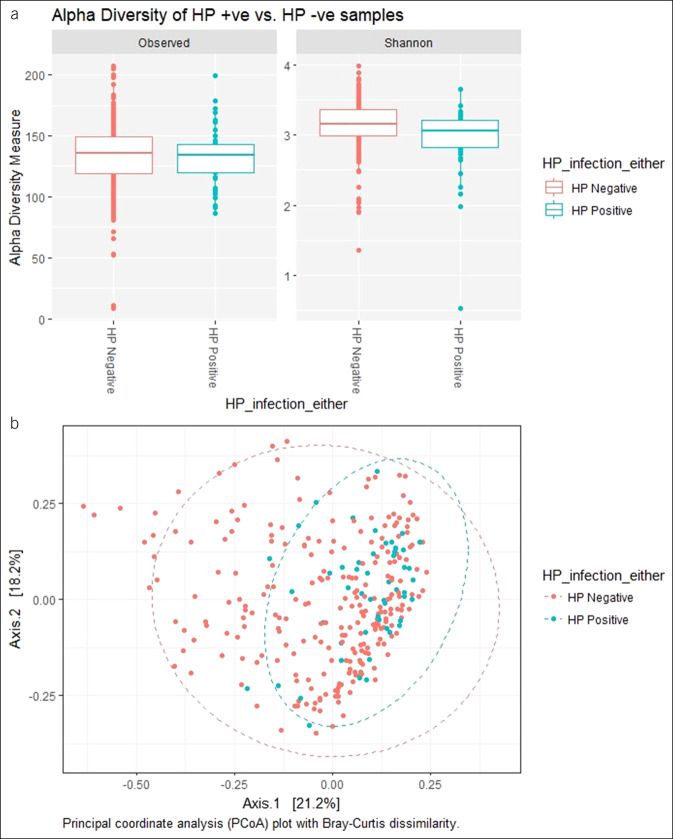
(**a**) Alpha diversity box plots of *H. pylori* status using observed (*P* = 0.39) and Shannon index (*P* = 0.001). Differences between the ranked mean alpha diversity estimates in each of the 2 categories were tested using the Wilcoxon test. (**b**) Principal coordinates analysis (PCoA) plots of Bray–Curtis distances showing a significant difference (*P* = 0.001, PERMANOVA test) between *H. pylori*–positive and *H. pylori*–negative samples. Red circles (positive) and turquoise circles (negative).

As expected from our low–*H. pylori* prevalence population, the proportion of *H. pylori* reads in all our sample groupings was very low (see Figure S7, Supplementary Digital Content 1, http://links.lww.com/CTG/A307). When samples were grouped as *H. pylori* positive or negative based on a positive result from either histology or serology, the *H. pylori* reads proportion remained low (Figure 5). We then assessed the variation of microbiota structure by *H. pylori* status by estimating microbial alpha diversity using the Shannon index. The *H. pylori*–negative group had a higher species evenness and richness compared with the *H. pylori*–positive group, as measured by the Shannon index (Figure 6a; observed [*P* = 0.39], Shannon [*P* = 0.001]; see Figure S9, Supplementary Digital Content 1, http://links.lww.com/CTG/A307). Similar results were obtained when using both histology and serology assays to define *H. pylori* status.

Assessment of structural similarities between the *H. pylori*–positive and *H. pylori*–negative groups was conducted based on the Bray–Curtis distance matrix and visualized using PCoA. Even though there was no distinct separation of samples by *H. pylori* status, *H. pylori*–positive samples tended to cluster together, although forming a subset of the larger *H. pylori*–negative cluster (Figure 6b), and a PERMANOVA test by *H. pylori* status showed a significant difference between the 2 groups (*P* = 0.001). A PCoA plot based on weighted UniFrac showed a similar pattern (see Figure S8, Supplementary Digital Content 1, http://links.lww.com/CTG/A307).

## DISCUSSION

We appraised differences in stomach microbial composition across the healthy and disease states in the largest study to date of the human gastric microbiota, with 316 individuals drawn from a low–*H. pylori* prevalence Western general population. Analysis of the community structure using the Shannon index suggested that the normal stomach appeared to have the highest microbial diversity, followed by antral chemical gastritis, whereas the atrophic gastritis and *H. pylori* gastritis groups had the lowest alpha diversity. In assessing whether *H. pylori* status affects bacterial diversity in the stomach, we found that the *H. pylori–*negative group had a higher species evenness and richness compared with the *H. pylori–*positive group, as measured by the Shannon index (observed [*P* = 0.39], Shannon [*P* = 0.001]).

Our findings of the taxonomic composition of the normal stomach at the phylum level with Firmicutes (42%), Bacteroidetes (24%), Proteobacteria (17%), Actinobacteria (7%), and Fusobacteria (6%) being dominant confirm previous studies on human gastric microbiota under healthy/normal conditions (37–39). At the genus level, 140 genera were identified, and *Streptococcus* (23%), *Prevotella* (22%), *Veillonella* (6%), *Fusobacterium* (5%), *Gemella* (4%), *Neisseria* (4%), and *Haemophilus* (4%) constituted the 7 top genera (68%).

Analysis of the diversity between the groups showed a clear separation of normal/antrum chemical gastritis samples and *H. pylori* gastritis/atrophic gastritis samples, as demonstrated by the PERMANOVA test, and the differential analysis using DESeq2 showed that the genus *Helicobacter* was a major component of the differences between all the groups. However, non–*H. pylori* bacteria have previously been reported (10,40) to contribute to the differences. Our results support this and remained so after the removal of the OTUs belonging to the genus *Helicobacter*, indicating that the differences are not solely driven by *H. pylori*. In the differential analysis, we still observed other genera, such as *Streptococcus*, *Bradyrhizobium*, *Propionibacterium*, and *Burkholderia* contributing to the between-group differences even in the absence of *H. pylori*. These findings may, however, be affected by the prevalence of *H. pylori* in the population where the study sample was drawn. Although our finding of the normal group being different from the *H. pylori* gastritis/atrophic gastritis groups is in agreement with Parsons et al. (10) in their study of 95 individuals, the removal of *H. pylori* from the analysis showed that the differences between normal and *H. pylori* gastritis were no longer significant, contrary to ours. Furthermore, they did not report any statistically significant difference between the normal group and atrophic gastritis group when *H. pylori* was included in the analysis. The differences in conclusions could partly be explained by the dominance of *H. pylori* sequences in the *H. pylori* gastritis group in their study (approximately 97% compared with 14% in our study). It is also possible that biopsy sampling might capture more mucosa-adherent bacteria compared with a brush sampling method, but a study (41) comparing brush and biopsy sampling methods of the ileal pouch for assessment of mucosa-associated microbiota in human subjects found that both the techniques provide similar assessments of the microbial community composition, with the brush sampling method having 3 main advantages, relatively more bacterial to host DNA, coverage of a larger surface area, and being less traumatic to the epithelium than a mucosal biopsy.

In the study by Parsons et al., the *H. pylori*–induced atrophic gastritis group showed a more complex microbial co-occurrence network compared with the nonatrophic *H. pylori* gastritis group (10). Our results support those findings and further show that the normal group network, with the highest average degree, is the most highly connected, followed by the atrophic gastritis group network, whereas the *H. pylori* gastritis group network had the least connection compared with the other groups. Communities with a high average degree would be assumed to harbor a high degree of functional redundancy, and therefore, changes in community composition may not correspond with changes in functional rates (42). Overall, co-occurrence analysis suggests the presence of different interactive patterns between these groups. Oral bacteria, depending on the stomach conditions, either pass through the stomach or have the potential to colonize it. Comparing our representative sequences to the Human Oral Microbiome Database (35), there were more positive co-occurrences of oral bacterial communities in the stomach because samples shifted away from the normal group network, although the strongest bacterial coexcluding interactions tended to be mostly between oral and nonoral bacteria among all the networks. Some species within the genera of the identified oral bacteria, e.g., *Peptostreptococcus*, *Prevotella*, *Centipeda*, *Actinomyces*, and *Atopobium*, are pathogenic (14,43,44) and might possibly contribute to cancer-promoting activities within the stomach early on in the Correa cascade.

In assessing whether *H. pylori* status affects bacterial diversity in the stomach, we found that the *H. pylori–*negative group had a higher species evenness and richness compared with the *H. pylori–*positive group, as measured by the Shannon index. Similar results were obtained when using both histology and serology assays to define *H. pylori* status. Even though there was no distinct separation of samples by *H. pylori* status, *H. pylori–*positive samples tended to cluster together, although forming a subset of the larger *H. pylori–*negative cluster, and the PERMANOVA test showed a significant difference between the 2 groups. A PCoA plot based on weighted UniFrac showed a similar pattern. This agrees with the previous findings from a 2008 Swedish study of 6 patients (38); however, data from a 2014 Malaysian study with 215 patients (18) found no differences. Noteworthy, however, is that the 2014 study (18) analyzed only the culturable bacteria from the stomach.

How colonization with *H. pylori* affects the gastric microbiota is not fully understood, but its density increases with worsening gastritis (45), probably allowing *H. pylori* to outcompete other bacteria (37,40). As hypochlorhydria increases because of chronic *H. pylori* infection (46), it probably exacerbates the colonization of the gastric mucosa by microbiota that would otherwise not be able to colonize this environment, such as the oropharyngeal bacteria. Alternatively, as the gastric mucosal barrier becomes more compromised because of *H. pylori* infection and subsequent inflammation, the resident non–*H. pylori* bacteria may develop pathogenic properties adhering to and even penetrating into the mucosa (47). The relationship between *H. pylori* and non–*H. pylori* bacteria in the pathogenesis of atrophic gastritis and gastric cancer needs further elucidation.

Some of the non–*H. pylori* bacteria that we have identified in the stomach potentially play a role in promoting inflammation and gastric carcinogenesis. Nitrosating bacteria, such as *Veillonella*, *Haemophilus*, *Staphylococcus*, *Streptococcus*, and *Neisseria* (48,49), can form N-nitroso compounds (NOC) that increase the risk of gastric cancer (48). The nitrosating capacity of NOC is acid–base equilibrium dependent and optimally functions at a low pH, and its proportion decreases with increasing pH in the stomach (50). Other stomach bacteria we identified, such as *Actinomyces*, *Corynebacterium*, *Haemophilus*, *Streptococcus*, and *Staphylococcus*, are known urease producers (51), a major inducer of innate immune response (52–54).

Some limitations of our study are that we used bacterial DNA as opposed to RNA; hence, we cannot distinguish whether the identified bacteria were metabolically active within the stomach. However, we observed an overlap of microbiota in our study compared with those identified in RNA-based studies (40,55), partly allaying this concern. It was not possible to identify which subjects with precancerous pathology would and would not progress to gastric cancer that may be microbiome dependent. Because we did not have access to information about the true history of *H. pylori* eradication in our cohort, we classified 20 participants with a histological diagnosis of post–*H. pylori* who were *H. pylori* positive on serology into the “post–*H. pylori* eradication/seropositive” group. This subgrouping might have led to some misclassification because it likely includes cases with overlooked *H. pylori* and a subgroup with past infection and thus presents another limitation of our study; studies based on posteradication therapy that succeeded will be better suited to study this subgroup.

The strengths of our study include the first evaluation of gastric microbiota in a random population-based sample, the large sample size (316 subjects), and the use of cytology brush samples. It is known that microbiota from tissue biopsy samples differ from gastric juice samples (56) irrespective of *H. pylori* infection. We hypothesize that the cytology brush approach, which collects cells by swabbing the gastric mucosa, provides a more representative view of the gastric mucosal microbiota through limiting the contamination of human DNA, a typical issue with tissue biopsy, although this hypothesis remains to be verified. Our use of a structured biopsy protocol and video-reviewed endoscopy process ensured standardized sample collection, and the objective phenotyping of subjects into disease groupings limits the scope for misclassification.

In conclusion, we show for the first time that the normal gastric mucosal microbiota based on brushings is very similar to the findings in antral chemical gastritis. Furthermore, the choice of a study reference group is important because we have demonstrated that the normal stomach is different from both nonatrophic *H. pylori* gastritis and atrophic gastritis that have been routinely used as reference groups in gastric microbiota studies. Previous work might therefore present a skewed picture of the baseline microbiota, and the choice of a comparison group will affect interstudy comparability. By comparing microbiota between the normal stomach to the early precancerous and other non-normal states in a low–*H. pylori* prevalence population, (57) we provide a clearer picture of the bacteria that potentially contribute to, or are part of, the dysbiosis associated with gastric carcinogenesis. An increased understanding of the role of non–*H. pylori* bacteria in the stomach mucosa may allow new approaches to prevention and treatment of disease in the future.

## CONFLICTS OF INTEREST

**Guarantor of the article:** Weimin Ye, MD, PhD.

**Specific author contributions:** N.N. conducted microbiome experiments and bioinformatics analysis, analyzed data, and wrote the manuscript; A.P. supervised biostatistical analysis data and critical revision of the manuscript for important intellectual content; A.F.A. supervised bioinformatics analysis and critical revision of the manuscript for important intellectual content; U.Z. coordinated laboratory experiments, the project, and critical revision of the manuscript for important intellectual content; A.A. coordinated the main project and critical revision of the manuscript for important intellectual content; M.V. performed serohistopathologic evaluation and critical revision of the manuscript for important intellectual content; N.J.T. guided analysis and critical revision of the manuscript for important intellectual content; L.A. codesigned the study, coordinated and collected study samples, and critical revision of the manuscript for important intellectual content; W.Y. designed and supervised the study, analyzed data, critical revision of the manuscript for important intellectual content, and obtained funding. All authors approved the final submitted draft.

**Financial support:** This study was supported by grants from the Swedish Research Council (2015-02625) and Swedish Cancer Society (2016-510). N.N. was partly supported by KID funding, and W.Y. was partly supported by a European Research Council consolidator grant (ERC-2015-CoG, no.: 682663). Funders had no involvement in the study design; in the collection, analysis, and interpretation of the data; in the writing of the report; and in the decision to submit the paper for publication.

**Potential competing interests:** None to report.

**Study Highlights**

**WHAT IS KNOWN**

✓ Non–*H. pylori* microbiota contribute to dysbiosis.
✓ *H. pylori* infection is a major cause of chronic gastritis cases that may eventually lead to gastric cancer through the Correa cascade in a subgroup.

**WHAT IS NEW HERE**

✓ The gastric mucosa–associated microbiota profile of the normal stomach is significantly different from that of patients with nonatrophic gastritis and atrophic gastritis. A true normal control group has rarely been included in past studies.
✓ Description of the normal microbiota in the healthy gastric mucosa from a general low–*H. pylori* prevalence population.
✓ The normal/healthy stomach microbiota are similar to the gastric microbiota in chemical gastritis.
✓ There is an increasing abundance of pathogenic bacteria because the gastric mucosa deviates from the normal state to potential early precancerous states.

## Supplementary Material

SUPPLEMENTARY MATERIAL
