# Supplementary material for: Gastric Microbiota in a Low–Helicobacter pylori Prevalence General Population and Their Associations With Gastric Lesions
Source: Clin Transl Gastroenterol. 2020 Jul 27;11(7):e00191. doi: 10.14309/ctg.0000000000000191 (PMC7431247; doi:10.14309/ctg.0000000000000191)
Supplement: SUPPLEMENTARY MATERIAL [file ct9-11-e00191-s008.docx]

# **Supplementary section**

# Gastric microbiota in a low-*Helicobacter pylori* prevalence general population and their associations with gastric lesions

## Methods supplementary section


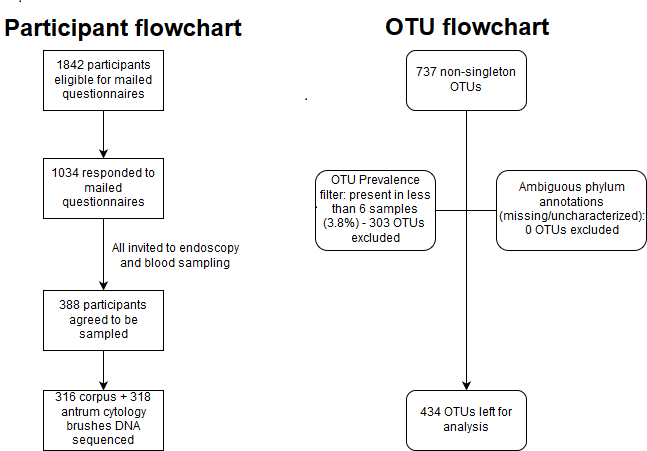


**Figure S0:** Flowchart diagrams for participant and OTU selection within this study.

### DNA extraction

The endoscopy brushes were transferred to 1.5ml Eppendorf safe-lock tubes using DNA-free forceps, 400µl 1X PBS added to each tube, and vortexed using the ELMI Intelli Mixer RM-2 using mode UU RPM 99 for 5 minutes. The tubes were then centrifuged (Eppendorf centrifuge 5427 R) at 8,000 rpm for 5min before repeating procedure to wash the brush for a second time. The two solutions from the brush wash were pooled into a new 1.5ml tube and the brush tip removed before centrifuging at 13,200 rpm for 18 minutes. The supernatant was gently removed and discarded followed by addition of 200µl of 20mg/ml lysozyme lysis buffer (20 mM Tris-HCl, pH 8.0; 2 mM sodium EDTA; 1.2% Triton X-100; Nuclease-free water) to each tube and mixing by pipetting up and down to re-suspend the pellet. The tube was then incubated for 1 hour at 37˚C with occasional light agitation. Using a DNA-free spatula, glass beads (0.5mm + 1mm, equal amounts) were added to each tube to reach no more than half of the lysis buffer, after which cell mechanical lysis was performed for 3 minutes using Bullet Blender® (BBX24. Next Advance. Inc., NY) at the speed 10. Approximately 200µl was subsequently transferred to new tubes for DNA extraction.

DNA was extracted by following the Mag Maxi Manual protocol of DNA Isolation Kit, Cat. No. 40403, LGC Genomics GmbH (Germany) with alterations in incubation times to 1 hour in the lysis, binding, and elution steps to obtain the maximum possible DNA yield from the samples after testing and discussions with the manufacturer.

### Library preparation and sequencing

The primers 341F (CCTACGGGNGGCWGCAG) and 805R (GACTACHVGGGTATCTAATCC) [24] targeting the V3-V4 regions of the bacterial 16S rRNA gene were used to generate PCR amplicons using KAPA HiFi HotStart ReadyMix (2X) (KAPA Biosystems, Kit Code KK2602) in replicates.

The reactions were carried out in a GeneAmp™ PCR System 9700 cycler (Applied Biosystems. CA) as follows: initial denaturation: 3 minutes at 95, then 10 seconds at 98 °C, 30 cycles of 20 seconds at 98 °C (denaturation), 30 seconds at 62 °C (annealing) and 30 seconds at 72 °C (extension) followed by 2 minutes at 72 °C as final extension. The replicates were pooled before the second barcoding PCR amplification which was carried out in a similar manner using dual indexing primers with similar conditions except that 10 cycles were used instead. In between the amplification cycles and before pooling sequencing libraries, the PCR products were purified using Agencourt AMPure XP (Beckman Coulter) following the manufacture’s recommendation. Amplicon fragment sizes and quantification were performed using Agilent 2100 Bioanalyzer (Agilent, Technologies, DNA 1000 LabChip kit) and Qubit® 2.0 Fluorometer (Invitrogen, Qubit-IT™ dsDNA HS Assay kit) respectively. Indexed samples were pooled at approximately equimolar amounts except for all negative controls from which 5ul was added to the pool. The pool was then sent to the National Genomics Infrastructure/Science for Life laboratories, Stockholm for sequencing on an Illumina MiSeq platform (Illumina Inc, USA) using a 2×300 bp paired end protocol (MiSeq Reagent Kits v3).

### Sample grouping (Serology and Histology)

The Normal/*H. pylori* negative gastritis group was defined as those who were histology normal and *H. pylori* negative on serology. *H. pylori* gastritis without corpus atrophy were those with a histological diagnosis of corpus and antrum *H. pylori* gastritis but with no corpus atrophy as well as a serology classification of *H. pylori* gastritis. Corpus atrophy (case) group was defined as those having corpus atrophy from either histology or serology. Antrum chemical gastritis was defined as those with a normal corpus diagnosis, antrum diagnosis of chemical gastritis, *H. pylori* negative on histology and not classified in other groups. Post *H. pylori* eradication/seropositive group were those with a histology corpus/antrum diagnosis of post *H. pylori* and were *H. pylori* positive on serology (Figure S13).

### Co-occurrence network analysis

SparCC uses centered log-ratio transformation to address data compositionality. SparCC was used with the following parameters (minimum correlation = 0.4, alpha = 0.05) to calculate correlations from the OTU count table. The MakeBootstraps command was used to generate 100 bootstrapped tables, which were in turn used to calculate correlations. The bootstrapped correlations were then used with the PseudoPvals command to generate two-tailed p-values for the SparCC correlations from the main analysis table.

### Prediction of metagenomics functional capacity

We used OTU representative sequences generated from Greengenes database (version 13.5) after performing a global alignment using USEARCH. Reads not aligning to the databases were discarded. Pathway predictions were conducted using Picrust [33]. Clusters of orthologous groups (COG) and Kyoto Encyclopedia of Genes and Genome (KEGG) orthology (KO) were generated after categorizing the predicted functional genes. Statistical comparisons between the analysis groups were then carried out using statistical analysis of taxonomic and functional profiles (STAMP) package [34]. Kruskal-Wallis H-test followed by Tukey-Kramer post-hoc test to identify differing pairs were performed with Benjamini-Hochberg False Discovery Rate (FDR) used for correcting multiple testing.

## Results supplementary section

**Sequencing control samples**

The positive control samples consisted of known mock bacterial communities of two types; even and staggered DNA concentrations. We recovered the expected mock communities (Figures S1, S2). The negative control samples consisted of low abundance taxa compared to experimental samples (Figures S3, S4).


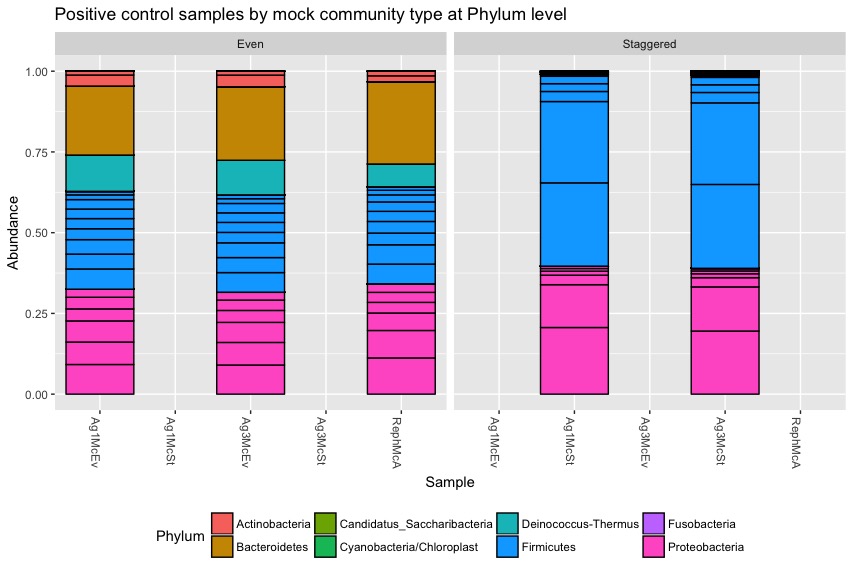


**Figure S1:** Bacterial mock community at the Phylum level. Even community mock samples are on the left panel and staggered community on the right panel.


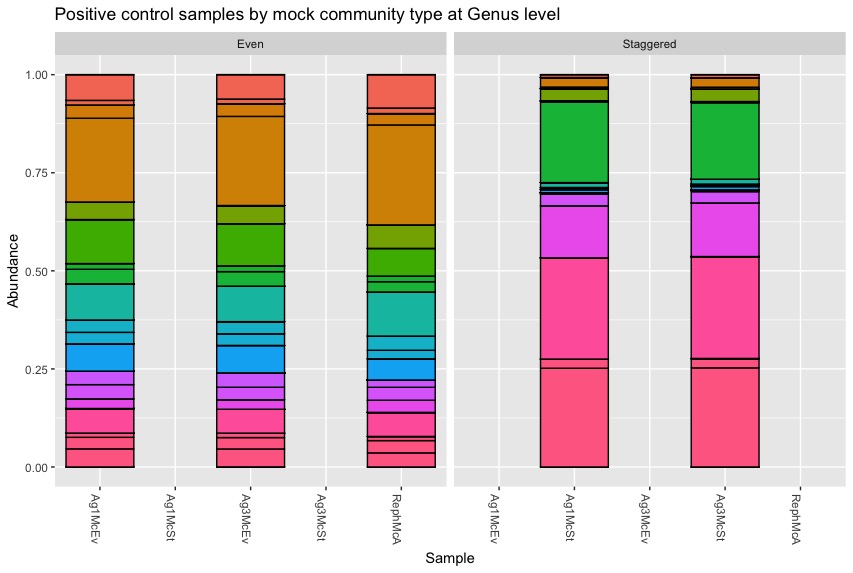


**Figure S2:** Bacterial mock community at the Genus level. Even community mock samples are on the left panel and staggered community on the right panel.


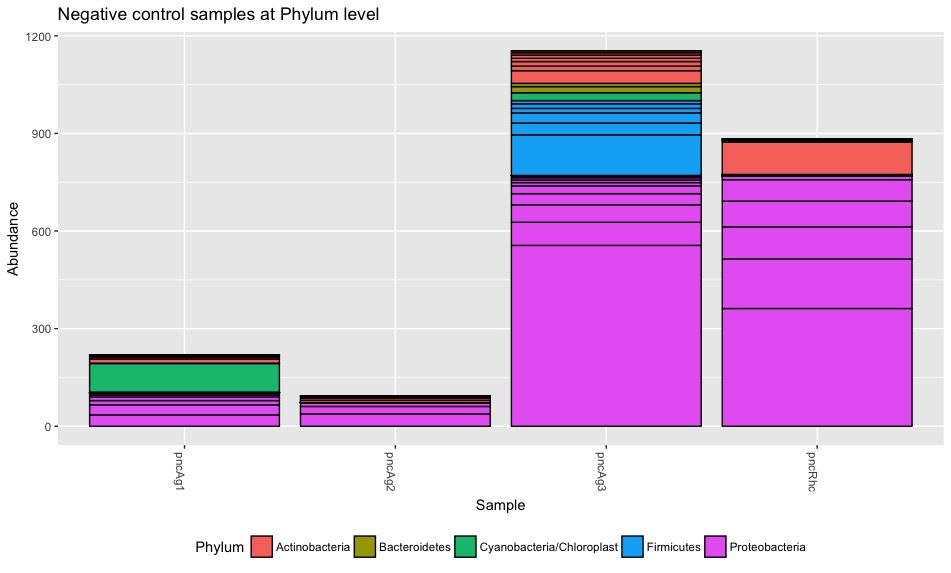
**Figure S3:** Phylum level abundance of the negative control samples.


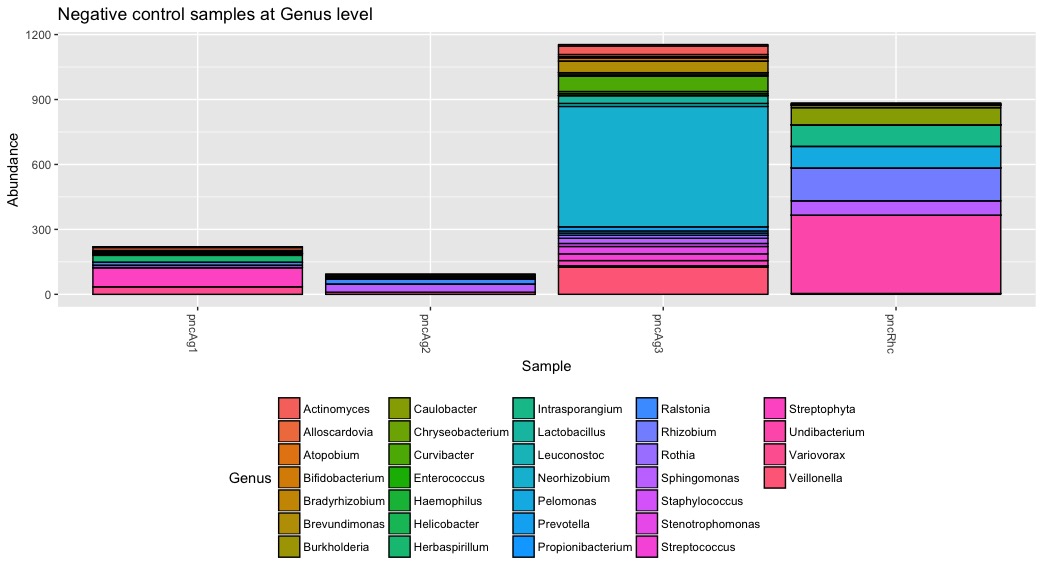


**Figure S4:** Genus level abundance of negative control samples.

### Similar microbiota in the stomach by anatomical positions

To assess the variation of microbiota structure by stomach anatomical sites (corpus and antrum), we estimated microbial alpha diversity (i.e. within sample diversity) using Shannon index. The corpus and antrum stomach anatomical sites had similar estimates of alpha diversity (Figure S5; Observed (p = 0.24), Shannon (p = 0.17)). We did not observe a difference in clustering by stomach sampling site when visualizing Bray Curtis distances in Principal Coordinates Analysis (PCoA) plot ((Figure S6), and a statistical test using PERMANOVA (Adonis test, in R Vegan package) did not show any significant difference in the two sites (R2 = 0.003, p = 0.05). Similarly, there were no enriched taxa found at one of the stomach anatomical site compared to the other that attained statistical significance (at 5% FDR level) using differential abundance testing (DESeq2) analysis, hence we focused the downstream analysis on the corpus samples.


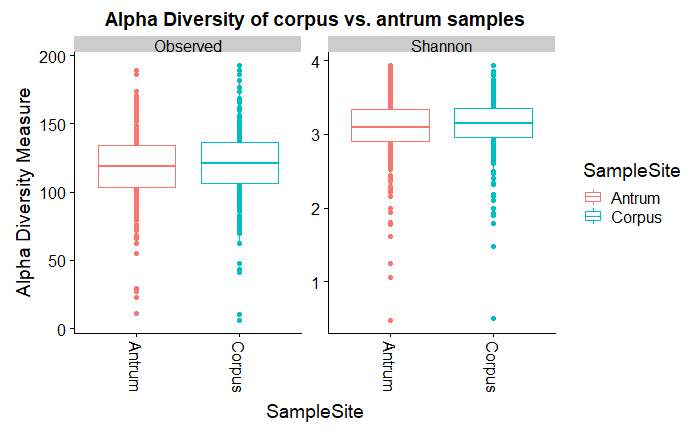


**Figure S5**: Alpha diversity box plots of corpus and antrum samples showing Observed richness (p = 0.24) and Shannon index (p = 0.17). Statistical differences between the ranked means in the two sampling sites for each of the alpha diversity parameters were tested using Wilcoxon test.


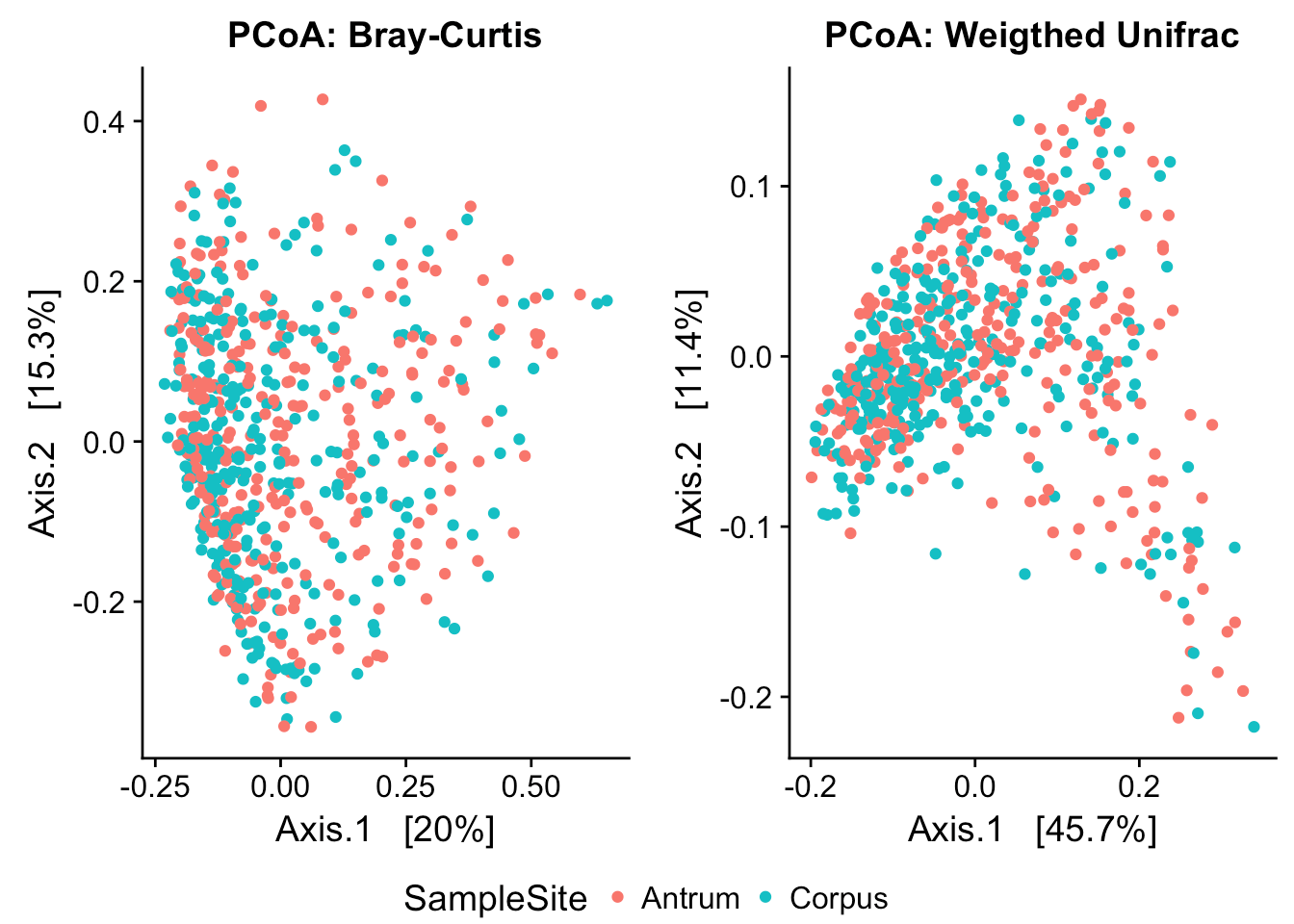


**Figure S6:** Principal Coordinates Analysis (PCoA) plots of Bray Curtis distances (The left panel) and weighted unifrac (right panel) showing no significant difference (p=0.05, Permanova test) between corpus and antrum samples


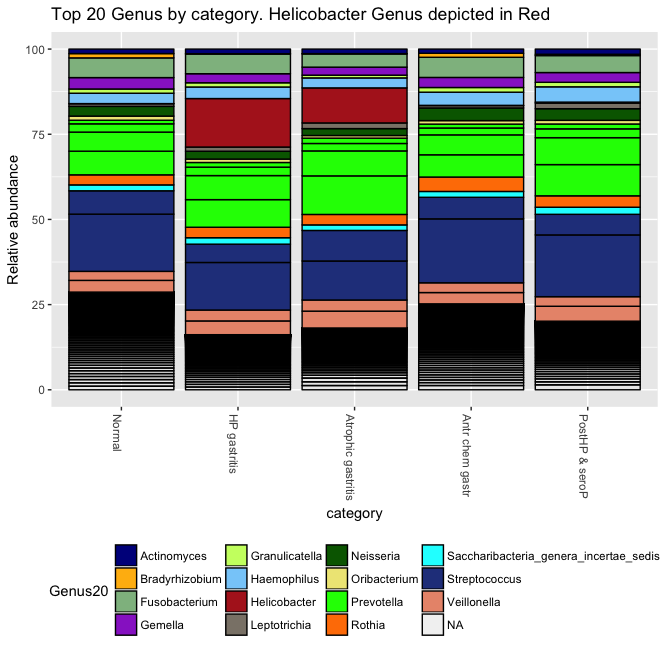


**Figure S7:** Proportion of *H. pylori* reads by analysis category, Normal = 0.03%, *Hp* gastritis = 14.24%, Atrophic gastritis = 10.28%, Antr chem gastr = 0.06%, PostHP & seroP = 0.28%


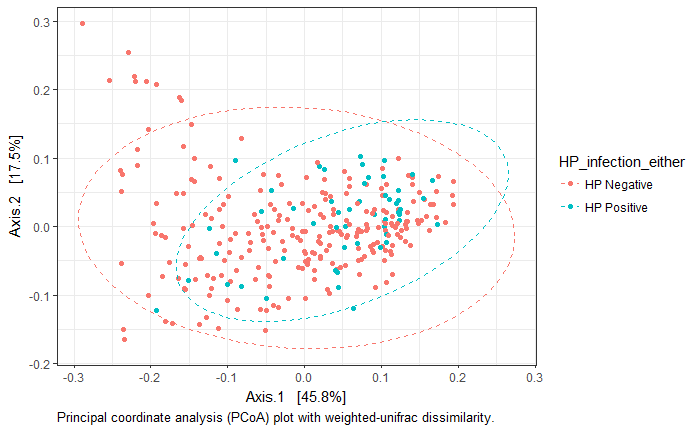


**Figure S8:** Clustering of samples by Helicobater status.


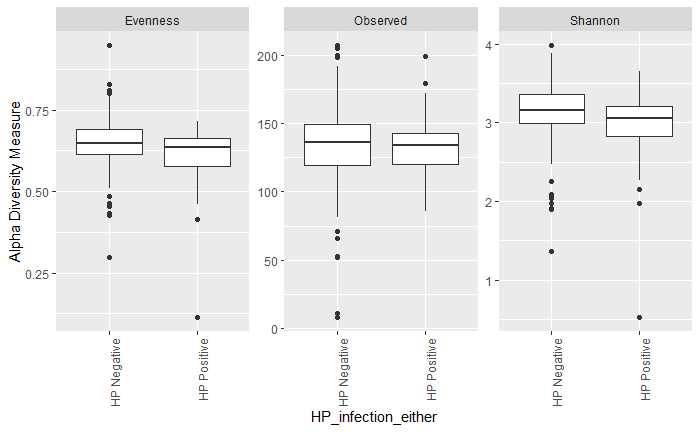


**Figure S9:** Pielou Evenness and Shannon alpha diversity by *H. pylori* status.

### Diversity between groups

Direct ordination using Canonical correspondence analysis (CCA), a constrained analysis that aids in elucidating the relationships between biological assemblages of species and their environment showed that the first axis, i.e. the main explainable variation in the species composition was negatively correlated with those classified as normal (ca. -4.5, strongly correlated given arrow length) and positively correlated with those classified as either *H. pylori* gastritis (ca. 9, strongly correlated) or atrophic gastritis (ca. 2, moderately correlated) (Figure S10). Those classified as post *H. pylori* eradication & serology positive were primarily related to the second axis (ca. 4.5). Redundancy analysis (RDA) that has the ability to detect any gradients unlike CCA gave similar results. Alternative methods to ordination were used to assess robustness of the results. Permutational multivariate analysis of variance (Permanova) derived from Bray-Curtis distance was used to test if samples differed between the main category groups (normal, atrophic gastritis, HP gastritis and antrum chemical gastritis) and we found that the distribution and abundances of microbiota composition were significantly different (p = 0.001) between the group. Similar results were found when using Permanova on both weighted and unweighted Unifrac distances. An investigation into whether the assumptions of the test were met showed that variance homogeneity assumptions we met (p = 0.09). Clustering by sample category was also visualized using weighted-unifrac dissimilarity in Principal Coordinates Analysis (PCoA) plot ((Figure S11).

### Analysis of group differences using OLGA grouping

In sensitivity analysis, we grouped participants using the Operative Link on Gastritis Assessment (OLGA) staging system. Stage 0 had 286 participants; Stage 1 had 11 participants while Stage 2 had 4 participants. To gain power in the comparisons we combined Stage 1 and Stage 2, and called this combined group Stage 1 (15 participants).

Permutational-multivariate analysis of variance (PERMANOVA) was used to test if samples differed between the OLGA staging system (Olga stage 0, and Olga Stages 1 while adjusting for age at endoscopy and batch effects (sequencing libraries). The distribution and abundances of microbiota composition were significantly different (p=0.002, permutations=999, R2=1.3%) between the groups.

Further, we conducted differential analysis using DESeq2 on raw prevalence filtered dataset to identify bacteria responsible for separation of the different groups at phylum and genus levels. We identified 9 genera that were differentially abundant between the two groups (Figure S17, Supplemental Digital Content Table S7). The genera that contributed to the differences were: *Lactococcus, Stomatobaculum, Corynebacterium, Burkholderia, Halomonas, Methylobacterium, Bradyrhizobium, Mesorhizobium,* and *Gluconacetobacter*.


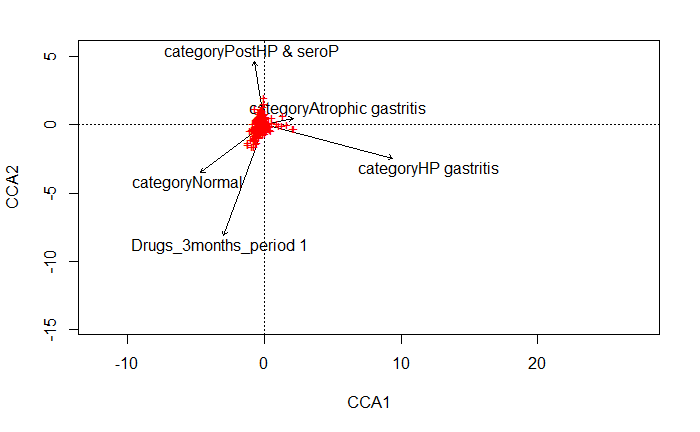


**Figure S10**: Canonical correspondence plot with the main environmental variables (category) and the covariate “drugs consumed within 3-months period” (p = 0.012) that was found to be significant during model selection. Red crosses represent bacterial taxa. The length of each arrow indicates the strength of the variable that explains the bacteria dispersion observed.


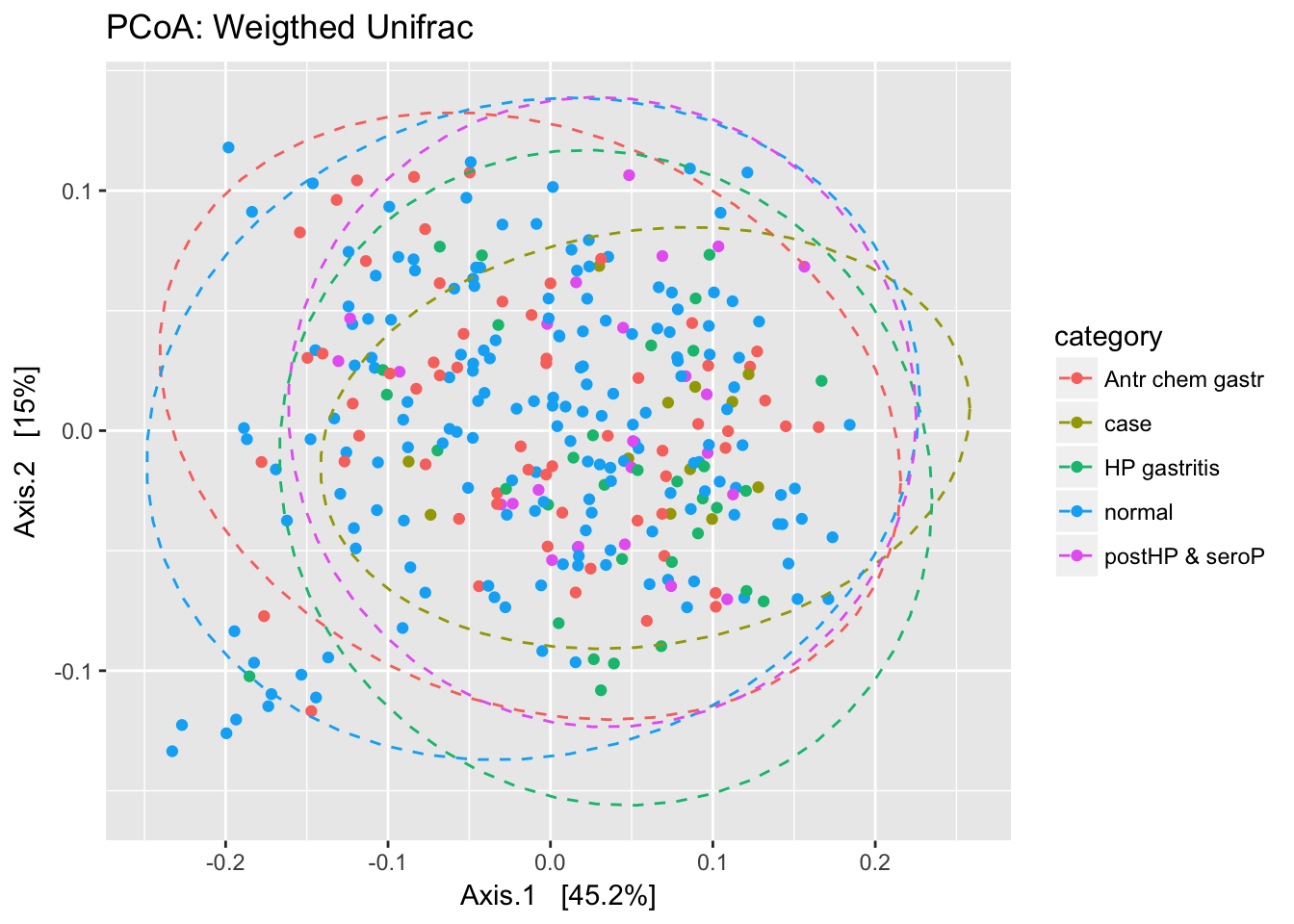


**Figure S11**: Principle coordinate analysis (PCoA) plot with weighted-unifrac dissimilarity. The category “case” refers to the group “atrophic gastritis”

**Microbial co-occurrence network analysis**

Co-occurrence analysis is used to explore interactions between microbes and environmental effects on their coexistence within biological communities. When two taxa co-occur or show a similar abundance pattern over multiple samples, a positive relationship is assumed while if they show mutual exclusion or anti-correlation, a negative one is predicted [36]. The derived co-occurrence patterns could indicate niche processes that drive coexistence and diversity maintenance within biological communities.

The full list of all the positively and negatively correlated genera is shown in Supplementary Table 2.

Among the strongest positively correlated genera within the normal group network were *Burkholderia* with *(Bradyrhizobium, Pelomonas, Acinetobacter* and *Propionibacterium), Pelomonas* with *Bradyrhizobium* and *Prevotella* with *Haemophilus*, while some the strongest negatively correlated genera were *Campylobacter* with (*Propionibacterium, Asticacaulis, Corynebacterium, Bradyrhizobium, Staphylococcus* and *Burkholderia*), *Bradyrhizobium - Prevotella,* and *Pelomonas - Haemophilus.*

The strongest positive associations within the *H. pylori* gastritis network at the genus level were *Prevotella* with (*Lachnoanaerobaculum*, *Oribacterium, Atopobium, Eubacterium), Stomatobaculum - Oribacterium, Eubacterium – Oribacterium* while the strongest negatively correlated genera were *Burkholderia – Haemophilus, Actinomyces* with *(Corynebacterium, Propionibacterium)* and *Filifactor – Rothia.*

Within the atrophic gastritis network the strongest positive associations at the genus level were *Neisseria* with (*Haemophilus, Peptostreptococcus, Gemella), Prevotella – Centipeda, Atopobium – Actinomyces* and *Streptococcus* - *Haemophilus* while the strongest negatively correlated genera were *Bifidobacterium* with *(Neisseria, Gemella), Prevotella* with *(Actinomyces, Bradyrhizobium), Streptococcus* with (*Acinetobacter, Stomatobaculum), Haemophilus – Acinetobacter, Catonella – Actinomyces.*

### Changes in pathways and genes in non-normal stomach from function prediction analysis

To link the bacterial phylogenies within the groups to potential microbial functions, we performed metagenomics function prediction using Picrust [33].

Those classified as normal or with antrum chemical gastritis could be distinguished from *H. pylori* gastritis and atrophic gastritis groups based on their predicted microbial functional profile. Some of the pathways that were statistically different (alpha = 0.05) between these groups were epithelial cell signaling in *H. pylori* infection (p =< 1e-15) (Supplementary Figure S12, upper panel), lipopolysaccharide biosynthesis proteins (p =< 1e-15) and bacterial toxins (p= 3.4e-15). Some of the outstanding genes showing differences (alpha = 0.05) between the normal/antrum chemical gastritis and *H. pylori* gastritis/atrophic gastritis groups were cytotoxin-associated gene A (CagA) pathogenicity proteins (Supplementary Figure S12, lower panel), vacuolating cytotoxin (Vac A) and urease genes. The full pathway and gene list are provided in the Supplementary Table 3 sheet 1 and sheet 2 respectively.


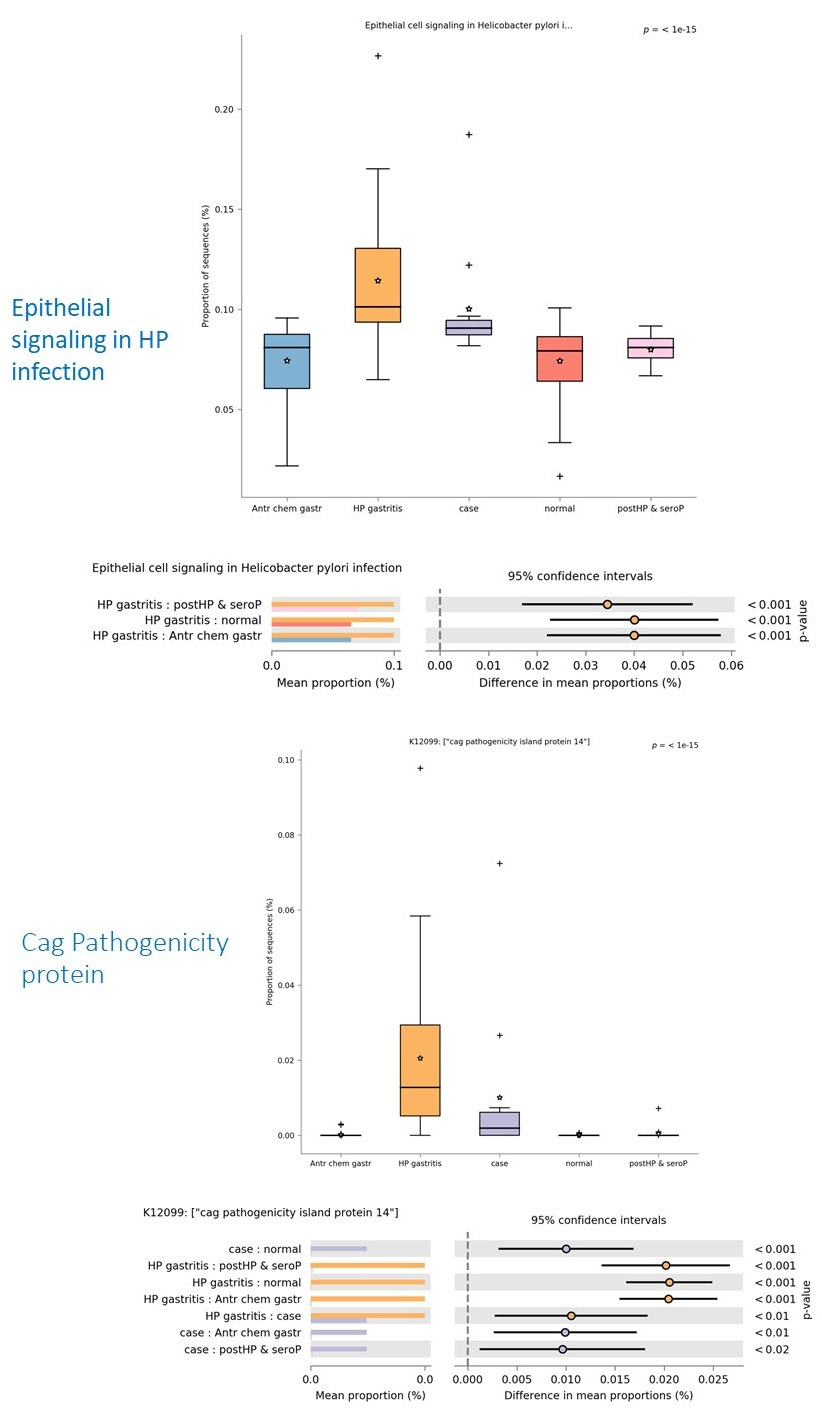


**Figure S12:** Functional prediction of pathways (upper panel) and genes (lower panel) that were found to be statistically different between the normal/antrum chemical gastritis groups vs. *H. pylori* gastritis/atrophic gastritis groups. For each panel the p-value (alpha = 0.05) of the box plots is shown at the top and the 95% confidence interval is shown at the bottom.


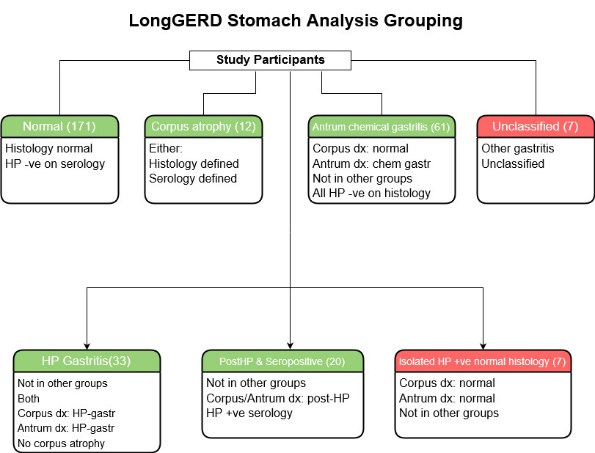


**Figure S13:** Study participants grouping for those with useable bacterial DNA sequences. NB: Groupings with red background were excluded from analysis while those with green background were included.


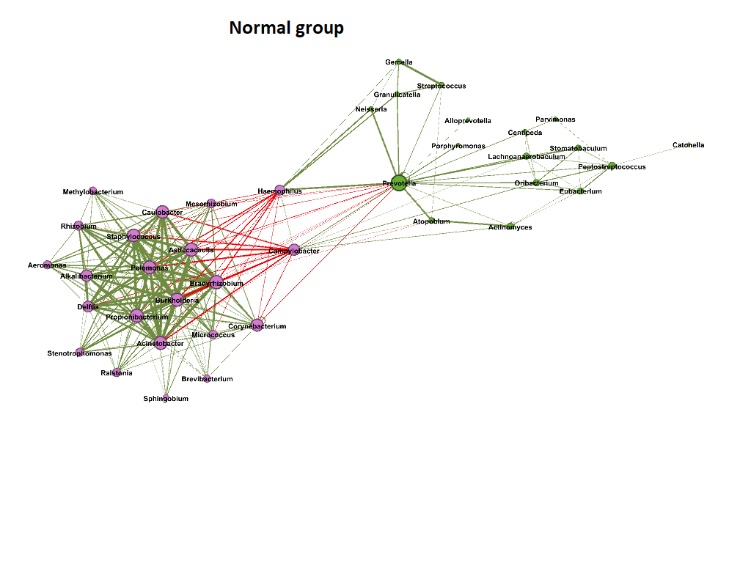


**Figure S14:** Normal group co-occurrence networks generated by SparCC. Nodes represent OTUs, with size reflecting the node’s degree (number of edges connected to a node), and node color corresponding to the communities to which the OTU belongs. Edges between nodes represent correlations between the nodes they connect, with edge width and shade indicating the correlation magnitude, and green and red colors indicating positive and negative correlations, respectively. Only edges corresponding to correlations whose magnitude is greater than 0.4 are drawn, and unconnected nodes are omitted


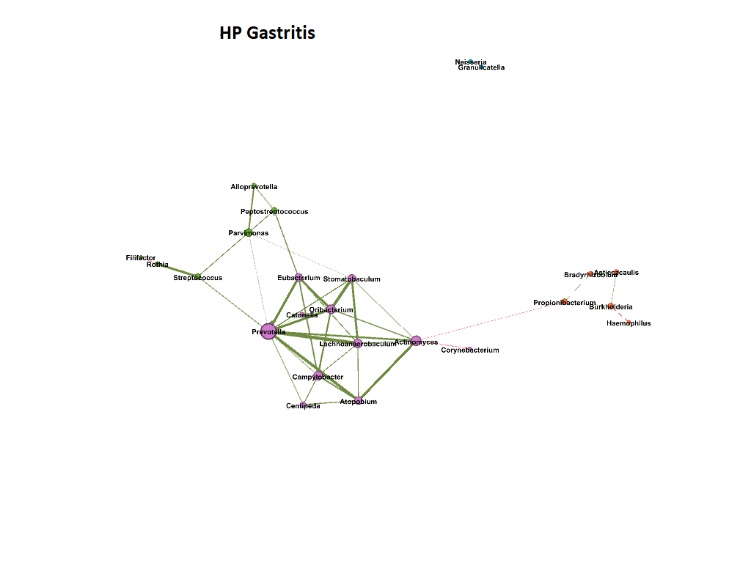


**Figure S15:** *H. pylori* gastritis group co-occurrence networks generated by SparCC. Nodes represent OTUs, with size reflecting the node’s degree (number of edges connected to a node), and node color corresponding to the communities to which the OTU belongs. Edges between nodes represent correlations between the nodes they connect, with edge width and shade indicating the correlation magnitude, and green and red colors indicating positive and negative correlations, respectively. Only edges corresponding to correlations whose magnitude is greater than 0.4 are drawn, and unconnected nodes are omitted.


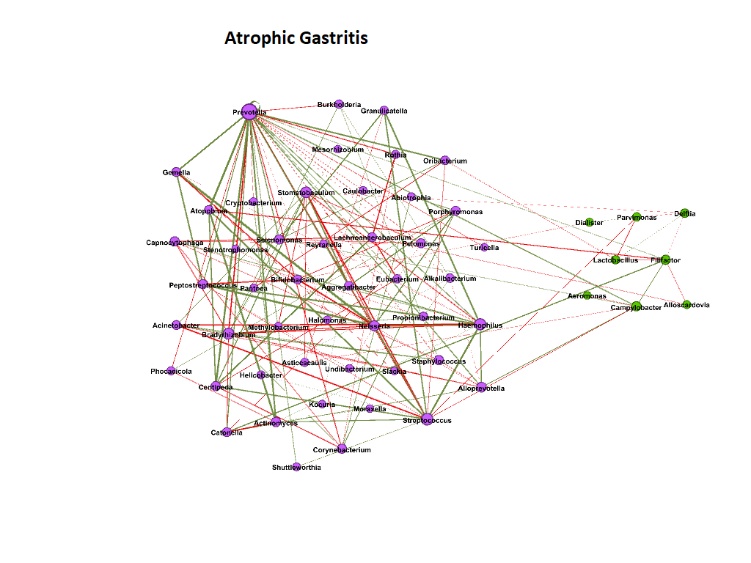


**Figure S16:** Atrophic gastritis group co-occurrence networks generated by SparCC. Nodes represent OTUs, with size reflecting the node’s degree (number of edges connected to a node), and node color corresponding to the communities to which the OTU belongs. Edges between nodes represent correlations between the nodes they connect, with edge width and shade indicating the correlation magnitude, and green and red colors indicating positive and negative correlations, respectively. Only edges corresponding to correlations whose magnitude is greater than 0.4 are drawn, and unconnected nodes are omitted.


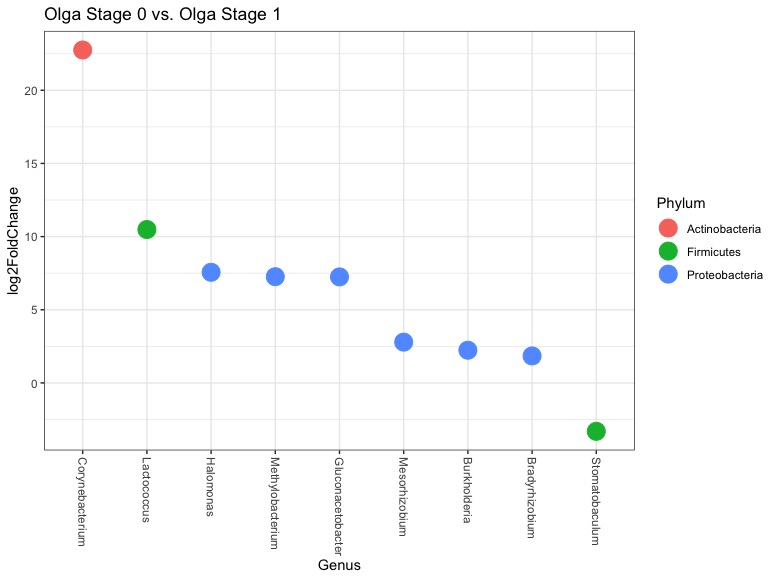


**Figure S17:** Differentially enriched OTUs between Olga Stage 0 – Olga Stage 1 at the genus and phylum level. These OTUs were among the most-significantly differentially abundant (alpha = 0.05, after multiple testing correction using Benjamini-Hochberg method) between the groups shown in the title of the respective plot
